# Factors Associated with Changes in Skeletal Muscle Mass in Medical Health Checkups

**DOI:** 10.3390/jcm14134683

**Published:** 2025-07-02

**Authors:** Saori Onishi, Akira Fukuda, Masahiro Matsui, Kosuke Ushiro, Tomohiro Nishikawa, Akira Asai, Soo Ki Kim, Sachiyo Yoshio, Hiroki Nishikawa

**Affiliations:** 1 Second Department of Internal Medicine, Osaka Medical and Pharmaceutical University, 2-7, Daigakumachi, Takatsuki 569-8686, Osaka, Japan; 2 Health Science Clinic, Osaka Medical and Pharmaceutical University, Takatsuki 569-8686, Osaka, Japan; 3 Department of Gastroenterology, Kobe Asahi Hospital, Kobe 653-8501, Hyogo, Japan; 4 Department of Human Immunology and Translational Research, National Institute of Global Health and Medicine, Japan Institute for Health Security, Shinjukuku 162-0052, Tokyo, Japan

**Keywords:** skeletal muscle mass, amount of change, sarcopenia, risk factor, metabolic disorder

## Abstract

**Background/Objectives:** To explore the factors associated with changes in skeletal muscle mass among Japanese health checkup subjects (5214 men and 6614 women). **Methods:** Fat-free index (FF index) was defined as FF mass divided by height squared (kg/m^2^). Change rate in FF index (kg/m^2^/year) was defined as [FF index (second time) − FF index (first time (i.e., baseline))]/interval between first and second times (years). Factors associated with change rate in FF index >0 kg/m^2^/year were primarily examined. **Results:** The average age, body mass index (BMI) were 52.4 years and 23.9 kg/m^2^ for men, and 50.5 years and 21.8 kg/m^2^ for women. In the multivariate analyses, age (*p* < 0.0001), body mass index (BMI, *p* < 0.0001), baseline FF index (*p* < 0.0001), waist circumference (*p* = 0.0365), fasting blood sugar (FBS, *p* = 0.0012), alanine aminotransferase (*p* < 0.0001) and alcohol intake were found to be significant in men, while BMI (*p* < 0.0001), baseline FF index (*p* < 0.0001), triglyceride (*p* = 0.0031), FBS (*p* = 0.0064) and alcohol intake were found to be significant in women. **Conclusions:** Lifestyle guidance from various aspects including metabolic factors may be important to maintain skeletal muscle mass.

## 1. Introduction

Skeletal muscles make up a large percentage of the human body, 30–40% [1]. One of the hallmarks of skeletal muscle is its robust regenerative capacity. Skeletal muscle tissue is composed of multinucleated giant cells called myofibers. Between the plasma membrane and basement membrane of muscle fibers, stem cells called mononuclear satellite cells are present [2]. Several to several dozen satellite cells are attached to each myofiber. It is known that cytokinesis does not occur in myofibers and that skeletal muscle tissue is regenerated by the action of satellite cells, which are stem cells [2]. Skeletal muscles have important functions such as movement, metabolism, and storage [3]. Skeletal muscle loss not only leads to decreased mobility and physical performance in daily activities but also causes metabolic disorders. Skeletal muscle loss is associated with the onset and severity of lifestyle-related diseases such as obesity and type 2 diabetes mellitus [4,5].

In recent years, academic research interest in skeletal muscle has increased, as exemplified by sarcopenia and frailty [6]. Maintaining skeletal muscle mass is critical for extending healthy life expectancy [4,5]. Fat-free index (FF index, FF mass (kg) divided by the square of height (m)) can be assessed with body composition analyzers widely available for home use [7]. FF index has been demonstrated to be a prognostic factor in various diseases such as malignancies, cardiovascular diseases, gastrointestinal diseases, and respiratory diseases [8,9,10,11,12,13,14,15]. It is widely known that skeletal muscle decreases with aging (i.e., primary sarcopenia) and underlying diseases such as malignancies, cardiovascular diseases, liver diseases, and respiratory diseases (i.e., secondary sarcopenia) [16].

However, there are numerous unknown factors that contribute to skeletal muscle gain, and there are also numerous risk factors for skeletal muscle loss among health checkup subjects [16]. Resistance training is known to lead to hypertrophy of skeletal muscle [17] and inhibit fat accumulation in skeletal muscle [18], while factors contributing to skeletal muscle gain other than resistance training are also clinically important in terms of improving sarcopenia and frailty and the associated extension of healthy life expectancy [19]. The purpose of this study was to explore the factors associated with changes in skeletal muscle mass among health checkup subjects.

## 2. Patients and Methods

### 2.1. Body Composition Analysis and Our Study Subjects

In this study, body composition of all subjects was examined at the Osaka Medical and Pharmaceutical University (OMPU) Health Sciences Clinic (OMPU attached facility). Between February 2022 and June 2024, all subjects agreed to the body composition analysis, and they were included in the current analysis. In the OPMU Health Sciences Clinic, bioimpedance analysis (TANITA corporation, Tokyo, Japan) has been performed for the evaluation of body composition. FF index was defined as FF mass divided by height squared (kg/m^2^). Total FF mass correlates extremely well with total body muscle mass [20]. Based on the previous reports, FF index < 18 kg/m^2^ in men and FF index < 15 kg/m^2^ in women was defined as skeletal muscle mass decline [20]. The annual rate of change in FF index (kg/m^2^/year) was calculated for a total of 11,828 cases with two body compositions performed from February 2022 to June 2024 in the OMPU health science clinic. Change rate in FF index (kg/m^2^/year) = [FF index (second time) − FF index (first time (i.e., baseline))]/interval between first and second times (years).

### 2.2. Type Classification According to the Daily Alcohol Consumed

In men, Type A was defined as never-drinkers, Type B as those who drank <210 g of ethanol equivalent in one week (i.e., <30 g/day), Type C as those who drank 210–420 g of ethanol equivalent in one week (i.e., 30–60 g/day), and Type D as those who drank >420 g of ethanol equivalent in one week (i.e., >60 g/day) [19,20]. In women, Type A was defined as never-drinkers, Type B as those who drank <140 g of ethanol equivalent in one week (i.e., <20 g/day), Type C as those who drank 140–350 g of ethanol equivalent in one week (i.e., 20–50 g/day), and Type D as those who drank >350 g of ethanol equivalent in one week (i.e., >50 g/day) [21,22].

### 2.3. Our Study and Ethical Approval

Factors linked to change rate in FF index >0 kg/m^2^/year (i.e., muscle mass increase) were primarily examined using univariate and multivariate analysis. We obtained ethical approval for the study from the ethics committee of OPMU hospital (approval no. 2025-021, approved on 21 April 2025). The protocol strictly observed all regulations of the Declaration of Helsinki. Consent from study subjects was waived due to the retrospective nature of this study.

### 2.4. Statistical Analysis

In the two-group comparison (continuous parameters), Student’s *t*-test or the Mann–Whitney U-test was applied, as applicable. In the multiple-group comparison (continuous parameters), analysis of variance (ANOVA) or Kruskal–Wallis test was applied, as applicable. In the group comparison (categorical parameters), Fisher’s exact test was applied. Factors with statistical significance for change rate in FF index > 0 kg/m^2^/year in the univariate analysis were subjected to multivariate regression analysis with multiple predictive variables using the least squares method to select candidate parameters. Unless otherwise mentioned, data are indicated as average (±standard deviation (SD)) value. We considered variables of *p* < 0.05 as statistically significant. JMP 17.0.0 software (SAS Institute, Cary, NC, USA) was used for statistical analyses.

## 3. Results

### 3.1. Baseline Characteristics

Baseline characteristics in this study (5214 men and 6614 women, clinical data at the time of the first body composition measurement) are shown in Table 1. The average (±SD) interval between the first and the second body composition measurement was 1.32 ± 0.46 years for men and 1.25 ± 0.43 years for women. The average (±SD) age, body mass index (BMI) and waist circumference (WC) were 52.4 ± 12.6 years, 23.9 ± 3.7 kg/m^2^ and 85.5 ± 9.9 cm for men, and 50.5 ± 11.5 years, 21.8 ± 3.7 kg/m^2^ and 78.0 ± 10.0 cm for women (all *p* values < 0.0001, men vs. women). WC of 85 cm or more (diagnostic criteria for metabolic syndrome in men) was found in 51.5% (2682 subjects: missing data for WC, *n* = 5) of men. WC of 90 cm or more (diagnostic criteria for metabolic syndrome in women) was found in. 11.8% (780 subjects: missing data for WC, *n* = 7) of women [23]. In terms of daily alcohol intake, type A/B/C/D was found in 1968 (37.7%)/2310 (44.3%)/493 (9.5%)/443 (8.5%) in men, and 4125 (63.7%)/1936 (29.3%)/401 (6.1%)/152 (2.3%) in women (*p* < 0.0001). The baseline average (±SD) FF index was 18.5 ± 1.5 kg/m^2^ in men and 15.1 ± 1.0 kg/m^2^ in women (*p* < 0.0001). The average (±SD) change rate in FF index was −0.0005 ± 0.48 kg/m^2^/year in men and 0.0028 ± 0.40 kg/m^2^/year in women (*p* = 0.2883). Change rate in FF index >0 kg/m^2^/year was found in 2550 subjects (48.9%) in men and 3258 subject (49.2%) in women. The percentage of baseline FF index <18 kg/m^2^ in men (i.e., skeletal muscle mass decline) was 38.2% (1990/5214), while the percentage of FF index <15 kg/m^2^ in women (i.e., skeletal muscle mass decline) was 45.8% (3032/6614).

### 3.2. Comparison of Change Rate in FF Index According to Age

**Figure 1:**
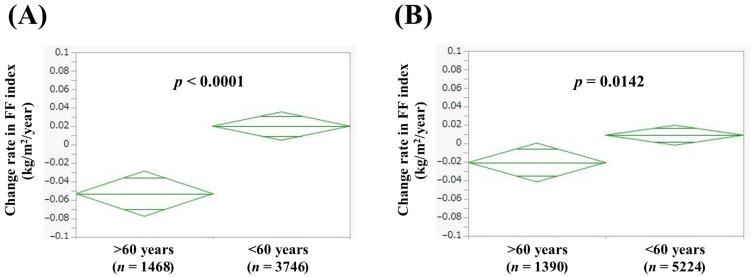
Change rate in FF index according to age in men (**A**) and women (**B**). Change rate in FF index (kg/m^2^/year) = [FF index (second time) − FF index (first time (i.e., baseline))]/interval between first and second times (years).

In men aged 60 years or more (*n* = 1468) and less than 60 years (*n* = 3746), the average (±SD) change rate in FF index was −0.053 ± 0.45 kg/m^2^/year and 0.020 ± 0.49 kg/m^2^/year (*p* < 0.0001, Figure 1A). In women aged 60 years or more (*n* = 1390) and less than 60 years (*n* = 5224), the average (±SD) change rate in FF index was −0.021 ± 0.39 kg/m^2^/year and 0.0090 ± 0.40 kg/m^2^/year (*p* = 0.0142, Figure 1B).

### 3.3. Comparison of Change Rate in FF Index According to BMI

**Figure 2:**
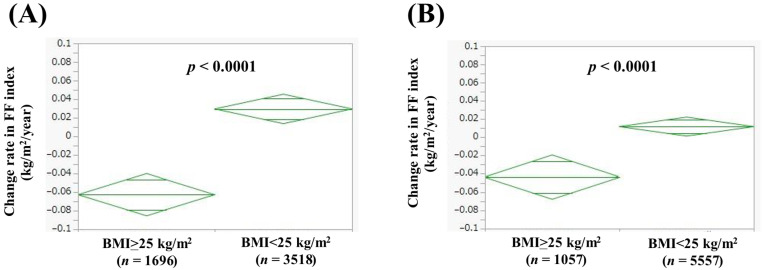
Change rate in FF index according to body mass index (BMI) in men (**A**) and women (**B**). Change rate in FF index (kg/m^2^/year) = [FF index (second time) − FF index (first time (i.e., baseline))]/interval between first and second times (years).

In men with BMI > 25 kg/m^2^ (*n* = 1696) and BMI < 25 kg/m^2^ (*n* = 3518), the average (±SD) change rate in FF index was −0.063 ± 0.57 kg/m^2^/year and 0.030 ± 0.43 kg/m^2^/year (*p* < 0.0001, Figure 2A). In women with BMI > 25 kg/m^2^ (*n* = 1057) and BMI < 25 kg/m^2^ (*n* = 5557), the average (±SD) change rate in FF index was −0.044 ± 0.45 kg/m^2^/year and 0.012 ± 0.39 kg/m^2^/year (*p* < 0.0001, Figure 2B).

### 3.4. Comparison of Change Rate in FF Index According to WC

**Figure 3:**
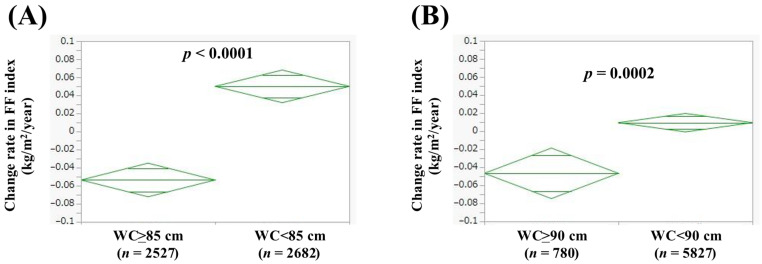
Change rate in FF index according to waist circumference (WC) in men (**A**) and women (**B**). Change rate in FF index (kg/m^2^/year) = [FF index (second time) − FF index (first time (i.e., baseline))]/interval between first and second times (years).

In men with WC >85 cm (*n* = 2527) and WC < 85 cm (*n* = 2682), the average (±SD) change rate in FF index was −0.054 ± 0.53 kg/m^2^/year and 0.050 ± 0.43 kg/m^2^/year (*p* < 0.0001, Figure 3A). In women with WC > 90 cm (*n* = 780) and WC < 90 cm (*n* = 5827), the average (±SD) change rate in FF index was −0.047 ± 0.45 kg/m^2^/year and 0.0094 ± 0.39 kg/m^2^/year (*p* = 0.0002, Figure 3B).

### 3.5. Comparison of Change Rate in FF Index According to Baseline FF Index

**Figure 4:**
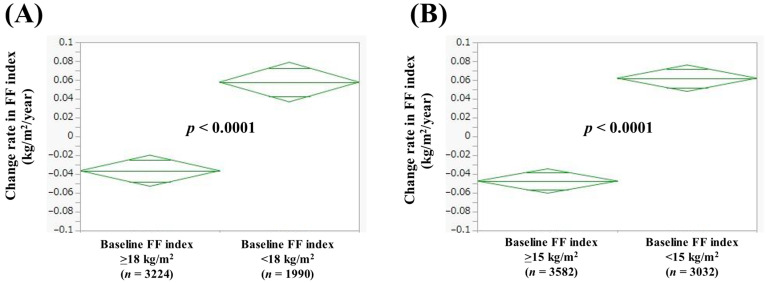
Change rate in FF index according to baseline FF index in men (**A**) and women (**B**). Change rate in FF index (kg/m^2^/year) = [FF index (second time) − FF index (first time (i.e., baseline))]/interval between first and second times (years).

In men with baseline FF index > 18 kg/m^2^ (*n* = 3224) and baseline FF index < 18 kg/m^2^ (*n* = 1990), the average (±SD) change rate in FF index was −0.036 ± 0.50 kg/m^2^/year and 0.058 ± 0.44 kg/m^2^/year (*p* < 0.0001, Figure 4A). In women with baseline FF index > 15 kg/m^2^ (*n* = 3582) and baseline FF index < 15 kg/m^2^ (*n* = 3032), the average (±SD) change rate in FF index was −0.047 ± 0.45 kg/m^2^/year and 0.062 ± 0.31 kg/m^2^/year (*p* < 0.0001, Figure 4B).

### 3.6. Comparison of Change Rate in FF Index According to Alcohol Intake

**Figure 5:**
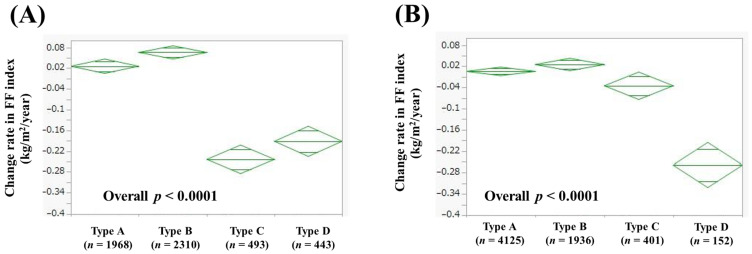
Change rate in FF index according to alcohol intake in men (**A**) and women (**B**). Change rate in FF index (kg/m^2^/year) = [FF index (second time) − FF index (first time (i.e., baseline))]/interval between first and second times (years). In men, Type A was defined as never-drinkers, Type B as those who drank <210 g of ethanol equivalent in one week, Type C as those who drank 210–420 g of ethanol equivalent in one week, and Type D as those who drank >420 g of ethanol equivalent in one week. In women, Type A was defined as never-drinkers, Type B as those who drank <140 g of ethanol equivalent in one week, Type C as those who drank 140–350 g of ethanol equivalent in one week, and Type D as those who drank >350 g of ethanol equivalent in one week.

In men, the average (±SD) change rate in FF index according to alcohol intake was 0.026 ± 0.45 kg/m^2^/year in Type A (*n* = 1968), 0.066 ± 0.45 kg/m^2^/year in Type B (*n* = 2310), −0.24 ± 0.31 kg/m^2^/year in Type C (*n* = 493), and −0.19 ± 0.72 kg/m^2^/year in Type D (*n* = 443) (*p* values: A vs. B, *p* = 0.0611; A vs. C, *p* < 0.0001; A vs. D, *p* < 0.0001; B vs. C, *p* < 0.0001; B vs. D, *p* < 0.0001; C vs. D, *p* = 0.0115; overall *p* < 0.0001, Figure 5A). In women, the average (±SD) change rate in FF index according to alcohol intake was 0.0056 ± 0.41 kg/m^2^/year in Type A (*n* = 4125), 0.025 ± 0.38 kg/m^2^/year in Type B (*n* = 1936), −0.035 ± 0.39 kg/m^2^/year in Type C (*n* = 401), and −0.26 ± 0.30 kg/m^2^/year in Type D (*n* = 152) (*p* values: A vs. B, *p* = 0.0744; A vs. C, *p* = 0.0495; A vs. D, *p* < 0.0001; B vs. C, *p* = 0.0056; B vs. D, *p* < 0.0001; C vs. D, *p* < 0.0001; overall *p* < 0.0001, Figure 5B).

### 3.7. Univariate and Multivariate Analyses of Factors Linked to Change Rate in FF Index > 0 kg/m^2^/Year

In men, age (*p* < 0.0001), BMI (*p* < 0.0001), baseline FF index (*p* < 0.0001), WC (*p* < 0.0001), alcohol intake (*p* < 0.0001), triglyceride (TG, *p* = 0.0184), fasting blood sugar (FBS, *p* < 0.0001), systolic blood pressure (sBP, *p* = 0.0007), diastolic BP (*p* = 0.0176), alanine aminotransferase (ALT, *p* < 0.0001), and gamma glutamyl transferase (*p* = 0.0119) were significant factors correlated with change rate in FF index > 0 kg/m^2^/year (Table 2). These eleven factors were subsequently entered into the multivariate regression analysis. In the multivariate analysis, age (*p* < 0.0001), BMI (*p* < 0.0001), baseline FF index (*p* < 0.0001), WC (*p* = 0.0365), FBS (*p* = 0.0012), ALT (*p* < 0.0001), Type C (*p* < 0.0001, Type A as a reference), and Type D (*p* = 0.0027, Type A as a reference) were found to be statistically significant (Table 3). Hazard ratios (HRs) and 95% confidence intervals (CIs) in each factor were demonstrated in Table 3.

In women, age (*p* < 0.0001), BMI (*p* < 0.0001), baseline FF index (*p* < 0.0001), WC (*p* = 0.0002), alcohol intake (*p* < 0.0001), TG (*p* = 0.0390), FBS (*p* = 0.0085), hemoglobin (*p* = 0.0014), serum albumin (*p* = 0.0369), and sBP (*p* = 0.0362) were significant factors correlated with change rate in FF index > 0 kg/m^2^/year (Table 4). These 10 factors were subsequently entered into the multivariate regression analysis. In the multivariate analysis, BMI (*p* < 0.0001), baseline FF index (*p* < 0.0001), TG (*p* = 0.0031), FBS (*p* = 0.0064), and Type D (*p* < 0.0001, Type A as a reference) were found to be statistically significant (Table 4). HRs and 95% CIs in each factor were demonstrated in Table 5.

## 4. Discussion

In the present study, we sought to investigate the factors associated with changes in skeletal muscle mass among health checkup subjects with a large sample size. The annual rate of change in FF index was calculated in cases in which body composition was measured twice at regular intervals, and the relationship with background factors was examined in an exploratory manner. The search for predictors of changes in skeletal muscle mass and its link to lifestyle guidance for patients is of great clinical importance [24], but to our knowledge, large-scale studies such as this one (i.e., a detailed study of the relationship between the amount of change in skeletal muscle mass and background factors) are rare. We thus believe the current results are worth reporting.

In this study, age, BMI, WC, alcohol consumption, ALT, FBS, and baseline FF index were independent factors contributing to the change rate in FF index > 0 kg/m^2^/year in the multivariate analysis in men. In other words, advanced age, obesity, heavy alcohol consumption, higher ALT level, higher FBS level, and higher baseline FF index are risk factors for skeletal muscle loss. In addition to age, factors related to fat and carbohydrate metabolism were shown to be risk factors for skeletal muscle loss. This is consistent with previous reports that metabolic abnormalities are risk factors for skeletal muscle mass loss [25,26]. Patients with obesity and type 2 diabetes are prone to “hyperinsulinemia” [27,28]. It has been reported that, along with increased blood insulin levels, increased amounts of “myostatin,” a molecule with inhibitory effects on skeletal muscle protein synthesis, lead to skeletal muscle loss [27,28]. Regarding alcohol consumption, the change rate in FF index was markedly lower in Type C and D subjects than in Type A and B subjects, suggesting the importance of providing guidance on alcohol consumption to subjects with metabolic and alcohol-related liver disease (Met-ALD) and ALD [19,20]. On the other hand, BMI, alcohol consumption, TG, FBS, and baseline FF index were independent factors contributing to the change rate in FF index > 0 kg/m^2^/year in the multivariate analysis in women. In other words, obesity, heavy alcohol consumption, abnormal fat metabolism, higher FBS level, and higher baseline FF index are risk factors for skeletal muscle loss. As in men, many metabolic factors were shown to be risk factors for skeletal muscle loss. However, unlike in men, age was not an independent factor. This is clinically important as it characterizes sex differences in skeletal muscle. One possible reason for these is that the average age of women with and without 30 min or more exercise per day was 53.9 years and 49.1 years (*p* < 0.0001) in this study. Physical activity among Japanese women may not decrease with age as much as it does among men. With regard to alcohol consumption, the change rate in FF index was markedly lower in Type D subjects than in Type A, B, and C (which is slightly different from the results in men), suggesting the importance of providing guidance on alcohol consumption to ALD subjects. It is consistent with previous reports that heavy drinking is a risk factor for sarcopenia [29]. The reason for sex difference of Type C (Figure 5) is unclear; however, the difference in cutoff values of alcohol intake in Type C between men and women (210–420 g of ethanol equivalent in one week in men and 140–350 g of ethanol equivalent in one week in women) may be related to the current results. Among the causes of cirrhosis, alcoholic cirrhosis has the highest complication rate of sarcopenia [30,31]. ALD elevates the risk of rapid muscle loss and mortality in cirrhotic patients [31]. Mechanisms of sarcopenia caused by heavy drinking have been reported, including direct impairment of skeletal muscle by acetaldehyde, skeletal muscle mass loss associated with increased myostatin from hyperammonemia, abnormal gut microbiota, starvation, growth hormone suppression, and sex hormone abnormalities [32,33]. In this study, the average BMI of male Type A, B, C, and D was 24.1 kg/m^2^, 23.7 kg/m^2^, 24.1 kg/m^2^, and 24.1 kg/m^2^, respectively, and the average serum albumin level of male Type A, B, C, and D was 4.4 g/dL, 4.4 g/dL, 4.4 g/dL, 4.3 g/dL, respectively. The average BMI of female Type A, B, C, and D was 21.8 kg/m^2^, 21.7 kg/m^2^, 21.6 kg/m^2^, and 21.6 kg/m^2^, respectively, and the average serum albumin level of female Type A, B, C, and D was 4.3 g/dL, 4.3 g/dL, 4.3 g/dL, and 4.3 g/dL, respectively. Thus, there are no notable differences in nutritional status between the four groups in both men and women. Combined impairment of skeletal muscle by mechanisms other than starvation may be the cause of skeletal muscle loss in heavy drinkers. On the other hand, no significant skeletal muscle loss was observed in Type A and B in both men and women, and it can be said that drinking is acceptable as long as the amount of alcohol consumed is within the range of no health hazard. Obesity was extracted as an independent risk factor for skeletal muscle loss in both men and women, an important result related to sarcopenic obesity [34]. Recently, diagnostic criteria for sarcopenic obesity were issued in Japan [34]. In both men and women, cases with preserved baseline FF index were a risk factor for skeletal muscle loss in our data. Although the reason for this is unknown, continuous management of diet and exercise is required to maintain muscle mass [35].

We must acknowledge several limitations to this study. First, this was a single facility-based cross-sectional observational study with a retrospective nature. Second, the analysis in this study is limited to skeletal muscle mass with missing data for grip strength, which is mandatory for sarcopenia. Third, this study was limited to the Japanese population, and it is not clear whether it is applicable to other ethnic groups. Fourth, clinical data for alcohol intake was based on self-report, potentially leading to bias. Fifth, the categorization of muscle mass change using FF index lacks clinically validated thresholds, which may limit interpretability and reproducibility. Finally, in individuals with marked edema, the bioimpedance analysis method may overestimate muscle mass, also creating bias [36]. Thus, our study results should be carefully interpreted. However, the present study revealed the involvement of various metabolic factors other than age as risk factors for skeletal muscle mass loss in medical health checkups.

## 5. Conclusions

In conclusion, clinicians should be aware of various metabolic factors to maintain skeletal muscle mass in Japanese health checkup recipients. Interventions to subjects with various metabolic factors may be necessary for preventing skeletal muscle mass loss.

## Figures and Tables

**Table 1:** Baseline features (*n* = 11828).

	Male (*n* = 5214)	Female (*n* = 6614)	*p* Value
Age (years)	52.4 ± 12.6	50.5 ± 11.5	<0.0001
BMI (kg/m^2^)	23.9 ± 3.7	21.8 ± 3.7	<0.0001
Alcohol intake, type A/B/C/D	1968/2310/493/443	4125/1936/401/152	<0.0001
WC (cm)	85.5 ± 9.9	78.0 ± 10.0	<0.0001
30 min or more exercise per day, yes/no/unknown	1537/3508/169	5215/1281/118	<0.0001
Hemoglobin (g/dL)	14.9 ± 1.1	13.1 ± 1.1	<0.0001
ALT (IU/L)	26.9 ± 19.8	17.4 ± 11.7	<0.0001
GGT (IU/L)	45.5 ± 54.3	23.2 ± 23.2	<0.0001
Serum albumin (g/dL)	4.4 ± 0.3	4.3 ± 0.2	<0.0001
Triglyceride (mg/dL)	118.9 ± 86.1	79.9 ± 50.6	<0.0001
FBS (mg/dL)	95.9 ± 19.6	88.6 ± 13.0	<0.0001
Uric acid (mg/dL)	6.2 ± 1.3	4.6 ± 1.0	<0.0001
eGFR (ml/min/1.73 m^2^)	70.6 ± 13.2	73.7 ± 13.2	<0.0001
sBP (mmHg)	122.6 ± 16.5	115.4 ± 17.2	<0.0001
dBP (mmHg)	79.3 ± 12.5	72.2 ± 12.0	<0.0001
Baseline FF index (kg/m^2^)	18.5 ± 1.5	15.1 ± 1.0	<0.0001
Change rate in FF index (kg/m^2^/year)	−0.0005 ± 0.48	0.0028 ± 0.40	0.2883

Data are shown as number or average (±standard deviation). BMI, body mass index; WC, waist circumference; ALT, alanine aminotransferase; GGT, gamma glutamyl transferase; FBS, fasting blood sugar; eGFR, estimated glomerular filtration rate; sBP, systolic blood pressure; dBP, diastolic blood pressure; FF index, fat-free mass divided by height squared. Alcohol intake in men: Type A, never-drinkers; Type B, <210 g of ethanol equivalent in one week; Type C, 210–420 g of ethanol equivalent in one week; Type D, >420 g of ethanol equivalent in one week. Alcohol intake in women: Type A, never-drinkers; Type B, <140 g of ethanol equivalent in one week; Type C, 140–350 g of ethanol equivalent in one week; Type D, >350 g of ethanol equivalent in one week.

**Table 2:** Univariate analysis of factors linked to change rate in FF index >0 kg/m^2^/year in males.

	Change Rate in FF Index > 0 kg/m^2^/year (*n* = 2550)	Change Rate in FF Index ≤ 0 kg/m^2^/year (*n* = 2664)	*p* Value
Age (years)	51.4 ± 12.6	53.4 ± 12.5	<0.0001
BMI (kg/m^2^)	23.7 ± 3.5	24.2 ± 3.8	<0.0001
Alcohol intake Type A/B/C/D	1023/1262/70/195	945/1048/423/248	<0.0001
WC (cm)	84.7 ± 9.6	86.2 ± 10.2	<0.0001
30 min or more exercise per day, yes/no/unknown	726/1744/80	811/1764/89	0.1050
Hemoglobin (g/dL)	14.9 ± 1.1	14.9 ± 1.1	0.7808
ALT (IU/L)	25.6 ± 17.9	28.2 ± 21.3	<0.0001
GGT (IU/L)	43.6 ± 54.8	47.3 ± 53.8	0.0119
Serum albumin (g/dL)	4.4 ± 0.3	4.4 ± 0.3	0.0974
Triglyceride (mg/dL)	116.1 ± 84.2	121.7 ± 87.9	0.0184
FBS (mg/dL)	94.3 ± 15.7	97.5 ± 22.7	<0.0001
Uric acid (mg/dL)	6.2 ± 1.2	6.2 ± 1.3	0.5697
eGFR (mL/min/1.73 m^2^)	70.9 ± 13.4	70.4 ± 13.0	0.2186
sBP (mmHg)	121.8 ± 16.5	123.3 ± 16.5	0.0007
dBP (mmHg)	78.9 ± 12.3	79.7 ± 12.5	0.0176
Baseline FF index (kg/m^2^)	18.3 ± 1.5	18.6 ± 1.5	<0.0001

FF, fat-free; BMI, body mass index; WC, waist circumference; ALT, alanine aminotransferase; GGT, gamma glutamyl transferase; FBS, fasting blood sugar; eGFR, estimated glomerular filtration rate; sBP, systolic blood pressure; dBP, diastolic blood pressure. Alcohol intake in men: Type A, never-drinkers; Type B, <210 g of ethanol equivalent in one week; Type C, 210–420 g of ethanol equivalent in one week; Type D, >420 g of ethanol equivalent in one week.

**Table 3:** Multivariate analysis of factors linked to change rate in FF index >0 kg/m^2^/year in males.

	HR	95% CI	*p* Value
Age (per one years)	0.989	0.984–0.994	<0.0001
BMI (per one kg/m^2^)	0.864	0.811–0.921	<0.0001
Alcohol intake			
Type A	1.000 (reference)		
Type B	1.100	0.971–1.246	0.1345
Type C	0.153	0.117–0.201	<0.0001
Type D	0.713	0.571–0.890	0.0027
WC (per one cm)	0.984	0.969–0.999	0.0365
ALT (per one IU/L)	0.993	0.989–0.996	<0.0001
GGT (per one IU/L)	1.001	0.999–1.002	0.1850
Triglyceride (per one mg/dL)	1.000	0.999–1.001	0.4706
FBS (per one mg/dL)	0.995	0.991–0.998	0.0012
sBP (per one mmHg)	0.998	0.992–1.004	0.4740
dBP (per one mmHg)	1.006	0.998–1.014	0.1238
Baseline FF index (per one kg/m^2^)	0.706	0.637–0.781	<0.0001

HR, hazard ratio; CI, confidence interval; FF, fat-free; BMI, body mass index; WC, waist circumference; ALT, alanine aminotransferase; GGT, gamma glutamyl transferase; FBS, fasting blood sugar; sBP, systolic blood pressure; dBP, diastolic blood pressure. Alcohol intake in men: Type A, never-drinkers; Type B, <210 g of ethanol equivalent in one week; Type C, 210–420 g of ethanol equivalent in one week; Type D, >420 g of ethanol equivalent in one week.

**Table 4:** Univariate analysis of factors linked to change rate in FF index >0 kg/m^2^/year in females.

	Change Rate in FF Index > 0 kg/m^2^/year (*n* = 3258)	Change Rate in FF Index ≤ 0 kg/m^2^/year (*n* = 3356)	*p* Value
Age (years)	50.0 ± 11.4	51.1 ± 11.6	<0.0001
BMI (kg/m^2^)	21.6 ± 3.6	22.0 ± 3.8	0.0001
Alcohol intake Type A/B/C/D	2048/995/196/19	2077/941/205/133	<0.0001
WC (cm)	77.5 ± 9.7	78.5 ± 10.3	0.0002
30 min or more exercise per day, yes/no/unknown	622/2588/48	659/2627/70	0.4929
Hemoglobin (g/dL)	13.1 ± 1.1	13.0 ± 1.2	0.0014
ALT (IU/L)	17.1 ± 10.9	17.8 ± 12.5	0.0517
GGT (IU/L)	23.1 ± 23.6	23.4 ± 22.7	0.5574
Serum albumin (g/dL)	4.3 ± 0.2	4.3 ± 0.2	0.0369
Triglyceride (mg/dL)	78.4 ± 48.7	81.5 ± 52.3	0.0390
FBS (mg/dL)	88.0 ± 11.1	89.3 ± 14.5	0.0085
Uric acid (mg/dL)	4.6 ± 1.0	4.6 ± 1.0	0.8223
eGFR (mL/min/1.73 m^2^)	73.7 ± 13.3	73.7 ± 13.2	0.9824
sBP (mmHg)	114.9 ± 16.7	116.0 ± 17.6	0.0362
dBP (mmHg)	71.9 ± 11.9	72.4 ± 12.1	0.0722
Baseline FF index (kg/m^2^)	15.0 ± 1.0	15.3 ± 1.0	<0.0001

FF, fat-free; BMI, body mass index; WC, waist circumference; ALT, alanine aminotransferase; GGT, gamma glutamyl transferase; FBS, fasting blood sugar; eGFR, estimated glomerular filtration rate; sBP, systolic blood pressure; dBP, diastolic blood pressure. Alcohol intake in women: Type A, never-drinkers; Type B, <140 g of ethanol equivalent in one week; Type C, 140–350 g of ethanol equivalent in one week; Type D, >350 g of ethanol equivalent in one week.

**Table 5:** Multivariate analysis of factors linked to change rate in FF index > 0 kg/m^2^/year in females.

	HR	95% CI	*p* Value
Age (per one years)	0.997	0.989–1.006	0.5546
BMI (per one kg/m^2^)	0.830	0.771–0.894	<0.0001
Alcohol intake			
Type A	1.000 (reference)		
Type B	1.049	0.881–1.249	0.5888
Type C	1.015	0.733–1.405	0.9286
Type D	0.096	0.038–0.242	<0.0001
WC (per one cm)	0.983	0.964–1.003	0.0922
Hemoglobin (per one g/dL)	1.047	0.971–1.128	0.2362
Serum albumin (per one g/dL)	1.164	0.812–1.670	0.4081
Triglyceride (per one mg/dL)	0.997	0.995–0.999	0.0031
FBS (per one mg/dL)	0.990	0.983–0.997	0.0064
sBP (per one mmHg)	1.002	0.997–1.008	0.3744
Baseline FF index (per one kg/m^2^)	0.515	0.432–0.612	<0.0001

HR, hazard ratio; CI, confidence interval; FF, fat-free; BMI, body mass index; WC, waist circumference; FBS, fasting blood sugar; sBP, systolic blood pressure. Alcohol intake in women: Type A, never-drinkers; Type B, <140 g of ethanol equivalent in one week; Type C, 140–350 g of ethanol equivalent in one week; Type D, >350 g of ethanol equivalent in one week.

## Data Availability

The original contributions presented in this study are included in the article. Further inquiries can be directed to the corresponding author(s).

## Abbreviations

FF; fat-free, OMPU; Osaka Medical and Pharmaceutical University, SD; standard deviation, BMI; body mass index, WC; waist circumference, TG; triglyceride, FBS; fasting blood sugar, BP; blood pressure, ALT; alanine aminotransferase, HR; hazard ratio, CI; confidence interval, ALD; alcohol related liver disease.
